# Chromosome-Scale Assembly of the Complete Genome Sequence of *Leishmania* (*Mundinia*) *martiniquensis*, Isolate LSCM1, Strain LV760

**DOI:** 10.1128/MRA.00058-21

**Published:** 2021-06-17

**Authors:** Hatim Almutairi, Michael D. Urbaniak, Michelle D. Bates, Narissara Jariyapan, Waleed S. Al-Salem, Rod J. Dillon, Paul A. Bates, Derek Gatherer

**Affiliations:** a Division of Biomedical and Life Sciences, Faculty of Health and Medicine, Lancaster University, Lancaster, United Kingdom; b Ministry of Health, Riyadh, Saudi Arabia; c Department of Parasitology, Faculty of Medicine, Chulalongkorn University, Bangkok, Thailand; Vanderbilt University

## Abstract

Leishmania (Mundinia) martiniquensis is a kinetoplastid parasite that was first isolated in 1995 on Martinique. We report the first complete genome for *Leishmania martiniquensis* from Asia, isolate LSCM1, strain LV760, which was sequenced using combined short-read and long-read technologies. This will facilitate greater understanding of the evolution of the geographically dispersed subgenus *Mundinia*.

## ANNOUNCEMENT

Parasites of the genus *Leishmania* infect a wide range of hosts, including humans. After malaria, leishmaniasis is the second most common cause of death due to parasitic infection (1), and it can be manifested in three main forms, i.e., visceral, cutaneous, and mucocutaneous. Around 100 countries have reported at least one of these three forms (2). The subgenus *Mundinia* (3) has been reported in dispersed locations worldwide, namely, Thailand (4), Martinique (5), Switzerland (6), Australia (7), and Ghana (8), but the only complete *Mundinia* genome, prior to the present work, is that of Leishmania (Mundinia) martiniquensis MAR LEM2494 (GenBank accession number GCA_000409445.2). We now report the complete genome of *L.* (*M.*) *martiniquensis* isolate Chiang Mai 1, strain LV760 (WHO code MHOM/TH/2012/LSCM1), which was originally obtained by bone marrow aspiration from a 52-year-old male patient from northern Thailand (9).

Genomic DNA isolation, cultivation, and extraction were performed using an *in vitro* culture system that had been developed previously for Leishmania (Mundinia) orientalis axenic amastigotes (10). Parasites were grown in Schneider's insect medium at 26°C as promastigotes and then in M199 medium supplemented with 10% fetal calf serum (FCS), 2% stable human urine, 1% basal medium Eagle vitamins, and 25 μg/ml gentamicin sulfate, with subpassage to fresh medium every 4 days to sustain parasite growth and viability. The extracted DNA concentration was assessed using a Qubit fluorometer, microplate reader, and agarose gel electrophoresis. All sequencing libraries were based on the same extracted DNA sample to avoid any inconsistency.

Short-read library construction and sequencing were contracted to BGI (Shenzhen, China) (DNBseq libraries producing paired-end reads [270 bp and 500 bp] using the Illumina HiSeq platform) and Aberystwyth University (Aberystwyth, UK) (TruSeq Nano DNA libraries producing paired-end reads [300 bp] using the Illumina MiSeq platform). We performed long-read library preparation and sequencing according to the Nanopore protocol (SQK-LSK109) on R9 flow cells (FLO-MIN106). Read quality was assessed using MultiQC (11).

We assembled the long reads with Flye (12), using default parameters, to generate chromosome-scale scaffolds. Then, using Minimap2 (13) and SAMtools (14), we mapped the short reads onto the assembled scaffolds to compensate for erroneous bases within the long reads and to create consensus sequences. After polishing of the assembly with Pilon (15), another round of consensus short-read mapping was performed. Then, we removed duplicated contigs and sorted the remainder according to length using Funannotate (16). Finally, we separated the chimeric sequences and performed scaffolding using RaGOO (17) with the Leishmania major Friedlin strain genome (GCA_000002725.2) (18) as a reference guide, aligning all 36 chromosomes for our assembly with the exception of six small contigs totaling 70,152 bp.

**FIG 1:**
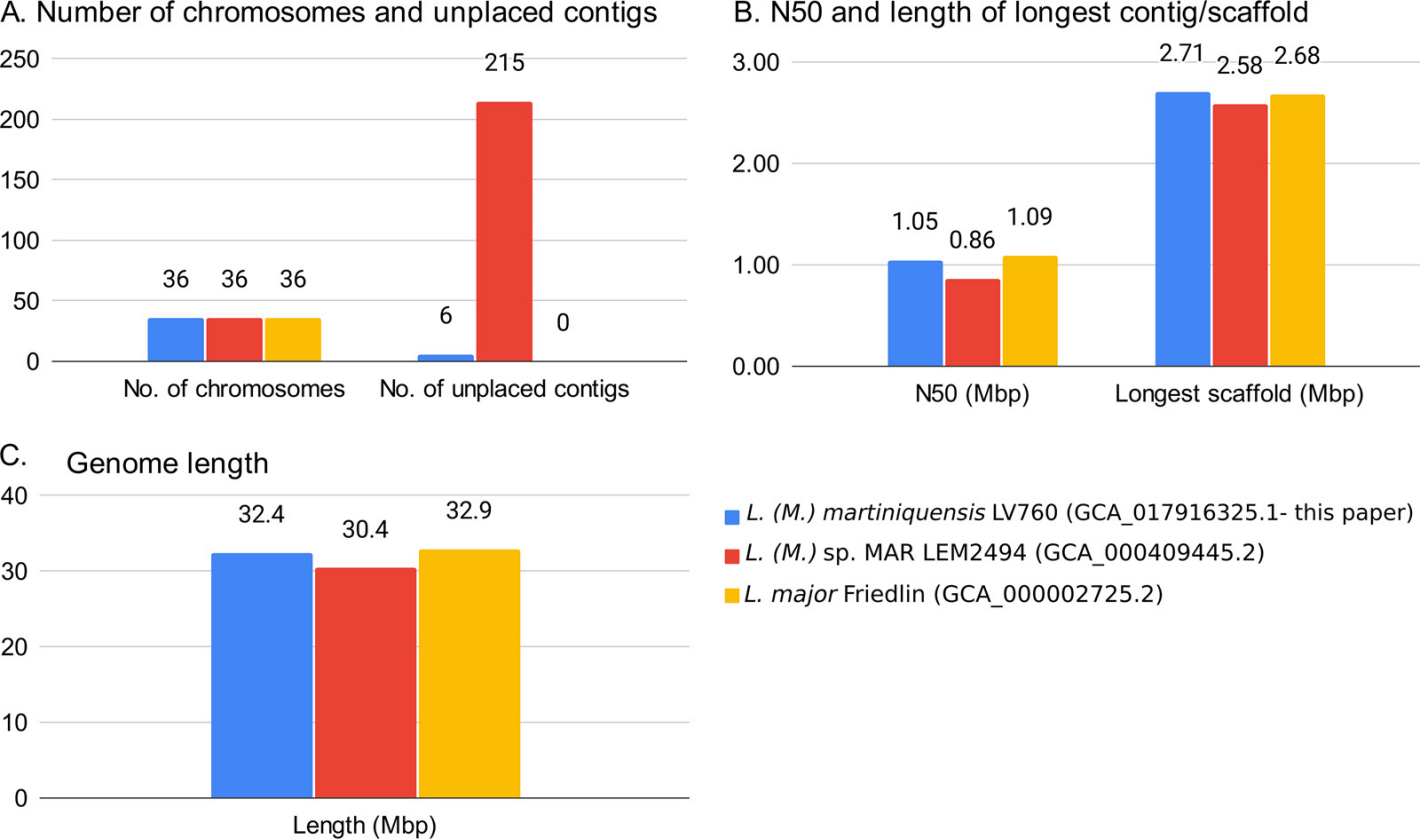
Assembly comparison of *L.* (*M.*) *martiniquensis* LV760 (this paper) with *L. martiniquensis* MAR LEM2494 and L. major Friedlin.

The analysis workflow for assembly and annotation, including software versions and parameters used, was performed using Snakemake (19) and is available online for reproducibility purposes (https://github.com/hatimalmutairi/LGAAP). Figure 1 compares our assembly with other complete genomes.

We assessed assembly completeness with BUSCO (20) using the lineage data set for the phylum *Euglenozoa*, containing 130 single-copy orthologues from 31 species, and we found 128 of the orthologues to be present (98.5% completeness). We carried out functional annotation and prediction using the MAKER2 (21) annotation pipeline in combination with AUGUSTUS (22) gene prediction software. Table 1 shows additional summary metrics for sequencing, assembly, and annotation.

**TABLE 1:** Summary of genome sequencing, assembly, and annotation metrics for *L.* (*M.*) *martiniquensis* LV760

Feature	Value
Total no. of reads	24,128,044
No. of MiSeq reads	4,462,344
No. of HiSeq reads	18,716,360
No. of MinION reads (read *N*_50_ [bp])	949,340 (15,090)
No. of bases (Gb)	19.24
Genome coverage (×)	277.9
Total no. of scaffolds	42
Genome size (bp)	32,413,670
*N*_50_ (bp)	1,046,741
GC content (%)	59.90
No. of Ns (% of genome)	50 (0.0002)
No. of genes	7,967
Gene density (no. of genes/Mb)	245.8
No. of exons	7,969
Mean gene length (bp)	1,857
Total length of coding sequences (Mb [% of genome])	14.80 (45.66)

### Data availability.

The assembly and annotations are available under GenBank assembly accession number GCA_017916325.1. The master record for the whole-genome sequencing project is available under accession number JAFEUZ000000000.1. Raw sequence reads are available under accession number PRJNA691531.
